# Age and Sex Differences in the Association between Serum Vitamin E Levels and Depressive Symptoms: Korea National Health and Nutrition Examination Survey

**DOI:** 10.3390/nu15081915

**Published:** 2023-04-15

**Authors:** Hyunwoo Jeong, Jae Won Oh, Nak-Hoon Son, San Lee

**Affiliations:** 1 Geumsan-gun Public Health Center, Geumsan 32726, Republic of Korea; 2 Department of Psychiatry, Yongin Severance Hospital, Yonsei University College of Medicine, Yongin 16995, Republic of Korea; 3 Department of Statistics, Keimyung University, Daegu 42601, Republic of Korea; 4 Department of Psychiatry and the Institute of Behavioral Science in Medicine, Yonsei University College of Medicine, Seoul 03722, Republic of Korea

**Keywords:** vitamin E, depressive symptoms, age, sex, KNHANES

## Abstract

Studies have reported inconsistent results regarding the relationship between serum vitamin E levels and depressive symptoms. Furthermore, the potential modulating role of age and sex has been underexplored. We conduct an age- and sex-stratified investigation of the association between serological vitamin E status and depressive symptoms in a large nationwide sample. Data from the Korean National Health and Nutrition Examination Survey were analyzed (*n* = 4448). The participants were stratified into four groups according to age (<65 vs. ≥65 years) and sex. Each group was divided into tertiles of vitamin E/total lipid ratio, and Patient Health Questionnaire-9 (PHQ-9) scores were compared among the tertiles via multivariable linear regression analyses. The relationship between dietary supplement use and the proportions of tertiles was assessed in each group. With the middle tertile as the reference group, the low tertile of vitamin E/total lipid ratio was associated with increased PHQ-9 scores in younger females and older males after adjusting for all covariates, while the high tertile showed no significant associations with PHQ-9 scores in any group. The low tertile was associated with increased adjusted mean PHQ-9 scores by 0.53 and 1.02 compared to the middle tertile in younger females and older males, respectively. Dietary supplement use was related to higher vitamin E/total lipid ratio in all four groups. In conclusion, younger females and older males with a low vitamin E status showed worse depressive symptoms. These individuals may benefit from dietary interventions to prevent depressive symptoms.

## 1. Introduction

Korea has undergone rapid economic growth and changes in social values over the last 50 years, which has caused mental health problems to become a national issue [1]. As a result, depression has become highly prevalent among Koreans; the prevalence of depressive symptoms is estimated to be 7% [2]. Depression causes impaired physical health and decreased quality of life; it inflicts a considerable burden on society [3,4]. Importantly, subthreshold depressive symptoms that are not severe enough to obtain a formal diagnosis can also cause significant harm in the same manner to that of clinical depression [5]. A preventive approach would be effective in tackling depressive symptoms in Korea because they are often not properly treated due to stigma on mental illnesses [6]. Therefore, identifying modifiable risk factors for depressive symptoms is an important task in Korea. Previous studies in Korea have identified several risk factors associated with depressive symptoms, including increased stress, poor health status, low income, and inadequate education [2,7]. However, there have been insufficient investigations on whether nutritional deficiencies are related to increased depressive symptoms in Koreans.

Nutritional deficiency can be a good target for modification because dietary supplement use is popular in Korea; a total of 62% of Korean adults use dietary supplements [8], and the most used ones are vitamin supplements [9,10]. A previous study suggested that multivitamin supplement use decreases the risk of depression in Korean older adults [11]. Many other benefits of dietary supplement use were demonstrated in large Korean samples, including higher rates of adequate micronutrient consumption, increased dietary total antioxidant capacity, and decreased risk of metabolic syndrome [12,13,14].

Vitamin E deficiency is highly prevalent in Korea. Approximately 90% of Korean adults have deficient or suboptimal levels of serum vitamin E [15], and only 13% consume adequate vitamin E above the recommended levels [16]. This could have negatively affected Koreans’ health, as vitamin E inadequacy has been associated with cognitive impairment, cardiovascular disease, cancer, infection, anemia, and poor pregnancy outcomes [17,18,19,20].

It has been suggested that depressive symptoms are associated with oxidative stress and inflammation [21]. For example, a positive correlation between oxidative stress markers and the severity of depressive symptoms has been reported [22,23], and a reduction in antioxidant enzyme activity in depressed patients has also been demonstrated [24]. Studies have also shown that depression is positively associated with pro-inflammatory markers [25,26].

Vitamin E is a fat-soluble antioxidant that also has anti-inflammatory effects [21]. Studies have shown that vitamin E supplementation reduces the levels of oxidative stress markers and increases antioxidant enzyme activity [27,28,29]. Vitamin E also reduces pro-inflammatory markers and suppresses enzymes related to inflammatory response [30,31,32].

Thus, it can be inferred that a low vitamin E status could be a risk factor for depressive symptoms. While inverse associations between dietary vitamin E intake and depression have been noted in several Asian studies [33,34,35,36], self-reported dietary intake may not be an accurate measure of an individual’s vitamin E status [37,38]; however, serum vitamin E concentration is a more accurate measure. Nonetheless, results from studies using serum levels of vitamin E have been limited and inconsistent. Two studies found no significant cross-sectional associations between serum vitamin E levels and depressive symptom severity [39,40], while several other studies reported that depressed individuals had lower serum vitamin E levels compared to non-depressed individuals [41,42,43].

The association between vitamin E status and depressive symptoms may vary according to age, since the risk factors and clinical presentations of late-life depression differ compared to depression in younger adults [44]. Notably, most previous studies on the relationship between serum vitamin E levels and depressive symptoms used samples of either younger adults [42,43,45] or older adults [39,46]. Prior studies have also indicated different associations between vitamin E and depression according to sex [33,39,46].

Given the paucity and inconsistency of the studies on the association between serum vitamin E levels and depressive symptoms, more research is needed; the potential modulating effect of age and sex on this association also needs further exploration. Accordingly, the present study conducts an age- and sex-stratified evaluation of the association between circulating concentrations of vitamin E and the severity of depressive symptoms using a large nationwide sample in Korea. This study also explores whether dietary supplement use is associated with vitamin E status, which might provide insights into the usefulness of dietary interventions. We hypothesized that vitamin E status derived from serum levels would be associated with depressive symptom levels and that this relationship would differ according to age and sex. We also hypothesized that dietary supplement use would be related to higher vitamin E status. With more than 4000 participants available for analysis, this is the largest serologic study of vitamin E and depressive symptoms to date.

## 2. Materials and Methods

### 2.1. Participants

Data were obtained from the 7th Korea National Health and Nutrition Examination Survey (KNHANES), which was conducted by the Korea Disease Control and Prevention Agency. The KNHANES is a nationwide population-based cross-sectional survey that assesses the health and nutritional status of Koreans; the resulting data inform the development and evaluation of health policies and programs in Korea. The survey is conducted annually throughout 192 regions in Korea, targeting approximately 10,000 individuals each year. The database is publicly available on the KNHANES website (http://knhanes.kdca.go.kr, accessed on 18 February 2023).

**Figure 1:**
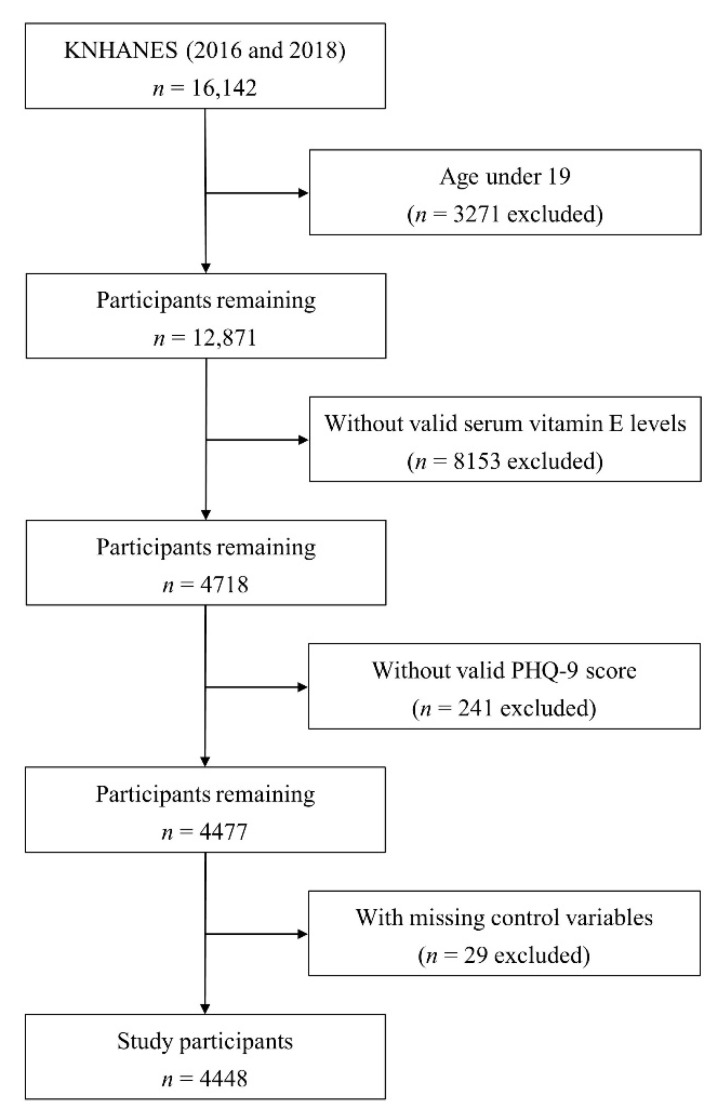
Flow diagram of the study participants. KNHANES, Korean National Health and Nutritional Examination Survey; PHQ-9, Patient Health Questionnaire-9.

As our study relied on data from the Patient Health Questionnaire (PHQ-9), which is administered biyearly, we investigated two years of data from the 2016 and 2018 KNHANES. Of the 16,142 participants included in these two years, people aged under 19 years were excluded (*n* = 3271). In addition, those without a valid serum vitamin E measurement (*n* = 8153), those without a valid PHQ-9 score (*n* = 241), and those missing covariate values (*n* = 29) were excluded. As a result, 4448 participants remained (Figure 1). In the original survey, all participants provided written informed consent and the survey was conducted according to the ethical standards of the Declaration of Helsinki. All study protocols and procedures of the KNHANES were reviewed and approved by the Korea Centers for Disease Control and Prevention Institutional Review Board (No. 2018-01-03-P-A), and the Institutional Review Board of Yongin Severance Hospital waived the requirements for approval.

### 2.2. Measures

#### 2.2.1. Patient Health Questionnaire-9

PHQ-9 is a nine-item depression questionnaire measuring the severity of depressive symptoms [47]. Each of the nine items is scored according to the frequency with which the patient has experienced that symptom over the past two weeks, from 0 (not at all) to 3 (nearly every day); total scores range from 0 to 27 [47]. The measure consists of the nine criteria used in the Diagnostic and Statistical Manual of Mental Disorders-IV [48] for diagnosing major depressive disorder (MDD) and was shown to be a reliable measure for assessing depressive symptom severity [47].

#### 2.2.2. Serum Vitamin E Levels, Serum Lipid Levels, and Adjustment of Vitamin E Levels According to Total Lipid Levels

Serum vitamin E levels were measured by high-performance liquid chromatography with flame ionization detector methods using the Agilent 1200 (Agilent Technologies Inc., Santa Clara, CA, USA). Total serum cholesterol levels and serum triglyceride levels were measured by the enzymatic method using the Hitachi Automatic Analyzer 7600-210 (Hitachi, Tokyo, Japan).

As vitamin E is carried by lipoproteins in circulation, serum vitamin E levels are positively affected by serum lipid levels [49]. A proper correction for this effect is required to accurately assess an individual’s vitamin E status [49]. In the present study, we controlled for this effect by calculating vitamin E/total lipid ratio; this has been suggested to be the most reliable estimate for evaluating vitamin E status in adults [50,51]. Total serum lipid levels were calculated from total serum cholesterol levels and serum triglyceride levels using the formula developed in a prior study [52], which was confirmed as highly accurate by another study [53].

#### 2.2.3. Covariates and Dietary Supplement Use Status

Based on clinical backgrounds and previous studies [54,55,56,57,58,59,60,61], a number of factors potentially associated with depressive symptoms were selected as covariates in the present study. Among sociodemographic variables, educational attainment, equalized household income, residential area, and living situation were included. Among clinical variables, alcohol use status, smoking status, chronic medical diseases, physical activity, and body mass index (BMI) were selected. Hypertension, diabetes mellitus, stroke, coronary heart disease, chronic kidney disease, cirrhosis, and chronic arthritis were classified as chronic medical diseases; participants were categorized as having 0, 1, or 2 or more of these diseases. Physical activity was measured by the Global Physical Activity Questionnaire [62]; we used World Health Organization (WHO) recommendations to assess its adequacy [63,64]. BMI was divided into four categories according to the Asian criteria developed by the WHO [65].

Dietary supplement use status was assessed by asking the participants whether they had taken dietary supplements regularly in the past year for a period of at least two weeks. Among the 4448 study participants, 560 participants did not answer or provided an invalid response. Therefore, exploratory analyses, including dietary supplement use data, were conducted with the remaining 3888 participants.

### 2.3. Statistical Analyses

The study participants were stratified into four groups according to age (younger: less than 65 years vs. older: 65 years or greater) and sex (male vs. female), and all analyses were performed separately in each group (i.e., younger males, younger females, older males, and older females). The age cut-off of 65 years was used because it is the traditionally used cut-off for late-life depression [39,66].

The baseline characteristics of the study population were assessed in each age and sex group. Group differences in continuous variables were tested using a one-way analysis of variance (ANOVA) with the post hoc Scheffe test or Welch’s ANOVA with the post hoc Games–Howell test as appropriate. For categorical variables, the four groups were compared using the chi-squared test or Fisher’s exact test as appropriate.

The current literature has not reached a consensus on cut-offs for defining normal serum vitamin E levels; accordingly, the need for developing age- and sex-specific cutoffs has been stressed [67]. In addition, the association between vitamin E and depressive symptoms may not be linear; non-linear relationships have been reported in other antioxidants [68]. For these reasons, we chose to divide each of the four age and sex groups into tertiles of vitamin E/total lipid ratio and compare PHQ-9 scores among the tertiles using linear regression analyses. The middle tertile (Tertile 2) was selected as the reference group, since they may represent individuals with relatively normal vitamin E status. In each age and sex group, two linear regression models were constructed to examine the relationships between vitamin E/total lipid ratio and PHQ-9 score: Model 1 did not adjust for any covariates, while Model 2 adjusted for all the covariates mentioned above. Adjusted mean PHQ-9 scores for each tertile were calculated from Model 2.

As an exploratory analysis, the odds ratio was calculated in each age and sex group to compare the odds of depressive symptoms according to serum vitamin E levels. The study participants were divided by PHQ-9 scores (<5 vs. ≥5) and serum vitamin E levels (<15 vs. ≥15 mg/L) for this analysis [69,70]. Adjusted odds ratio was also calculated in each group using multivariable logistic regression analysis, controlling for all the covariates mentioned above.

To explore whether dietary supplement use could affect an individual’s vitamin E status, the association between dietary supplement use status and the proportions of vitamin E/total lipid ratio tertiles was assessed using the Cochran–Armitage trend test in each age and sex group. In addition, the prevalence of dietary supplement use among all the study participants was calculated. All statistical analyses were performed using SAS software (version 9.4; SAS Institute, Cary, NC, USA), and figures were produced using R software (version 4.0.2) [71]. A *p*-value of <0.05 was considered statistically significant.

## 3. Results

### 3.1. Participants’ Baseline Characteristics

The baseline characteristics of the study participants are shown in Table 1. The four age and sex groups showed significant differences in all variables (*p* < 0.001), including vitamin E/total lipid ratio, PHQ-9 score, educational attainment, equalized household income, residential area, living situation, alcohol use status, smoking status, chronic medical disease, physical activity, and BMI. Among the four groups, vitamin E/total lipid ratio levels were the lowest in younger males (1.99 ± 0.54 mg/g) and the highest in older females (2.38 ± 0.85 mg/g). The levels of younger females (2.16 ± 0.56 mg/g) and older males (2.16 ± 0.66 mg/g) were in between. Post hoc group comparisons revealed that all group pairs had significantly different vitamin E/total lipid ratios (all *p* < 0.001), except for younger females and older males (*p* = 1.000). PHQ-9 scores of younger males (1.99 ± 3.12) and older males (2.11 ± 3.63) were relatively low, while those of younger females (2.87 ± 3.74) and older females (3.43 ± 4.68) were relatively high. Post hoc group comparisons showed that the PHQ-9 scores of younger and older males were not significantly different (*p* = 0.929), nor were scores of younger and older females (*p* = 0.072). All other group pairs had significantly different PHQ-9 scores (younger male vs. younger female: *p* < 0.001; younger male vs. older female: *p* < 0.001; older male vs. younger female: *p* = 0.001; older male vs. older female: *p* < 0.001). 

### 3.2. Association between Vitamin E/Total Lipid Ratio and Depressive Symptoms

Table 2 shows the results of the linear regression analysis between vitamin E/total lipid ratio tertiles and PHQ-9 scores in each age and sex group. In the unadjusted analyses (Model 1), the low tertile (Tertile 1) was associated with increased PHQ-9 scores in younger females (β = 0.08, *p* = 0.004) and older males (β = 0.16, *p* = 0.004). In contrast, the low tertile had no significant associations with PHQ-9 scores in younger males (β = 0.01, *p* = 0.834) and older females (β = 0.05, *p* = 0.349). The high tertile (Tertile 3) showed no significant associations with PHQ-9 scores in any age and sex group. After adjustments for the selected covariates (Model 2), the association between the low tertile and PHQ-9 scores remained significant in both younger females (β = 0.07, *p* = 0.008) and older males (β = 0.13, *p* = 0.018).

**Figure 2:**
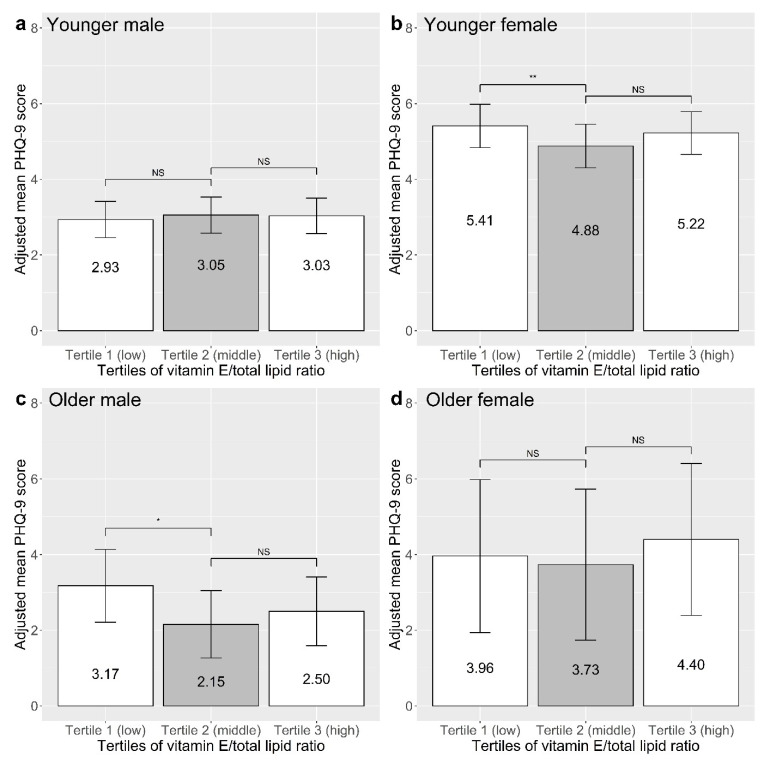
Levels of depressive symptoms by tertiles of vitamin E/total lipid ratio in (**a**) younger males, (**b**) younger females, (**c**) older males, and (**d**) older females. Mean PHQ-9 scores were adjusted for educational attainment level, equalized household income, residential area, living situation, alcohol use status, smoking status, number of chronic medical diseases, adequacy of physical activity, and BMI category. Group effects of Tertile 1 or 3 (white boxes) on PHQ-9 scores were examined via multivariable linear regression analyses. Tertile 2 (gray box) was set as the reference group for the analyses. Error bars indicate 95% confidence intervals. NS: not significant; * *p* < 0.05; ** *p* < 0.01. PHQ-9, Patient Health Questionnaire-9.

The adjusted mean PHQ-9 scores by tertiles of vitamin E/total lipid ratio are shown in Figure 2. The adjusted mean PHQ-9 scores and their 95% confidence intervals were as follows: younger males: Tertile 1: 2.93 [2.45, 3.41]; Tertile 2: 3.05 [2.57, 3.53]; Tertile 3: 3.03 [2.57, 3.50]; younger females: Tertile 1: 5.41 [4.84, 5.98]; Tertile 2: 4.88 [4.30, 5.45]; Tertile 3: 5.22 [4.66, 5.79]; older males: Tertile 1: 3.17 [2.21, 4.14]; Tertile 2: 2.15 [1.26, 3.05]; Tertile 3: 2.50 [1.59, 3.41]; older females: Tertile 1: 3.96 [1.94, 5.98]; Tertile 2: 3.73 [1.74, 5.73]; Tertile 3: 4.40 [2.39, 6.41].

In the exploratory analysis that calculated unadjusted and adjusted odds ratios, no significant association between vitamin E status and depressive symptoms was discovered in all age and sex groups.

### 3.3. Association between the Covariates and Depressive Symptoms

The association between the covariates and PHQ-9 scores in linear regression analyses (Model 2) are shown in Supplementary Table S1. Among younger males, those who received education up to middle school showed lower PHQ-9 scores than those who had attended only elementary school or less (β = −0.09, *p* = 0.008). The following equalized household income groups had lower PHQ-9 scores compared to that of the lowest quartile (Quartile 1): Quartiles 2, 3, and 4 in younger males (β = −0.17, *p* < 0.001; β = −0.24, *p* < 0.001; β = −0.27, *p* < 0.001, respectively) and younger females (β = −0.15, *p* < 0.001; β = −0.16, *p* < 0.001; β = −0.22, *p* < 0.001, respectively); Quartiles 3 and 4 in older males (β = −0.11, *p* = 0.046; β = −0.14, *p* = 0.019, respectively); and Quartile 3 in older females (β = −0.14, *p* = 0.008). Younger females who resided in rural areas had higher PHQ-9 scores compared to those in urban areas (β = 0.05, *p* = 0.017). Cohabiting younger males had lower PHQ-9 scores than those who lived alone (β = −0.09, *p* < 0.001). Smokers had higher PHQ-9 scores than non-smokers among younger males (β = 0.11, *p* < 0.001) and younger females (β = 0.15, *p* < 0.001). In all four age and sex groups, participants with two or more chronic medical diseases had higher PHQ-9 scores than those with no chronic medical disease (younger males: β = 0.10, *p* < 0.001; younger females: β = 0.10, *p* < 0.001; older males: β = 0.12, *p* < 0.042; older females: β = 0.14, *p* = 0.030). Older males who attained an adequate amount of physical activity had higher PHQ-9 scores than those who did not (β = 0.11, *p* = 0.023).

### 3.4. Association between Dietary Supplement Use and Vitamin E/Total Lipid Ratio

The proportions of vitamin E/total lipid ratio tertiles according to dietary supplement use status are presented in Supplementary Figure S1. Dietary supplement users had higher vitamin E/total lipid ratios compared to non-users in all four age and sex groups (*p*-trend < 0.001 in all four groups). The prevalence of dietary supplement use among all the study participants was 50.6%.

## 4. Discussion

The present study assessed vitamin E status from serum concentrations and investigated its association with depressive symptoms. In younger females and older males, the low tertiles of vitamin E/total lipid ratio were associated with worse depressive symptoms, independently of sociodemographic and clinical factors, whereas no such association was found in younger males and older females. However, high tertiles of vitamin E/total lipid ratio were not significantly associated with depressive symptoms in any group. Dietary supplement use was related to an increased vitamin E/total lipid ratio.

Specifically, the low vitamin E/total lipid ratio tertile was associated with increased adjusted mean PHQ-9 scores by 0.53 and 1.02 in younger females and older males, respectively. Although these differences in depressive symptom severity may seem small, the low baseline depressive symptom levels of the current study population should be considered when interpreting these results. A recent study that explored the minimal clinically important difference (MCID) of PHQ-9 scores discovered that individuals with milder baseline depressive symptom severity had smaller MCID estimates; MCID was smaller than 1 for baseline PHQ-9 scores lower than 7 [72]. This suggests that a slight change in depressive symptom levels can still be clinically significant for individuals with low baseline depressive symptom levels. Given that the mean PHQ-9 scores of younger females and older males were both lower than 3 in our study, the PHQ-9 score differences we found in terms of the relationship to vitamin E/total lipid ratio could be large enough to have some clinical significance.

As low tertiles of vitamin E/total lipid ratio were associated with increased depressive symptom severity in the present study, we suggest that low vitamin E status could represent a weaker antioxidative defense in the brain. The fact that this association was only found in younger females and older males may be explained by the action of sex hormones. Evidence from prior studies has indicated that both androgens and estrogens have antioxidant properties in the brain [73,74,75]. Serum androgen levels have been demonstrated to be negatively associated with serum vitamin E levels in males [76,77], while a positive association between serum estrogen levels and serum vitamin E levels has been reported in females [78]. Thus, in a vitamin-E-deficient condition, the weakened antioxidative capacity due to a low vitamin E status could be counteracted in males (by higher serum androgen levels) but augmented in females (by lower serum estrogen levels).

In younger adults, the action of sex hormones is more prominent since the serum levels of both androgens and estrogens are higher than those among older adults [79,80]. Therefore, there is a stronger counteracting effect of androgens and augmenting effect of estrogens in younger males and younger females, respectively. In addition, younger females had greater depressive symptom scores compared to younger males, providing more room for an antidepressant-like action of vitamin E. These factors may explain why a low vitamin E status was associated with worse depressive symptoms in younger females but not in younger males. This finding is in line with a meta-analysis that reported that dietary vitamin E intake among depressed females was lower than that among the controls, but that no such difference was found in males [33]. As most studies included in this meta-analysis used samples primarily comprising individuals aged less than 65 years, its results may be interpreted in line with the younger participants of the present study.

In contrast, the action of sex hormones is relatively less prominent in older adults. This means that the counteracting effect of androgens is weaker in older than younger males, and the augmenting effect of estrogens is weaker in older than younger females. This might explain why the association between vitamin E status and depressive symptoms was significant in older but not younger males, and vice versa among females. This finding is consistent with two previous studies that explored the relationship between serum vitamin E levels and depression in older adults. One study reported that higher baseline serum vitamin E levels were associated with a slower progression of depressive symptoms in older males but not in older females [39]. Another study demonstrated that serum vitamin E levels were lower in older males with MDD compared to healthy older males, but such association was not observed in older females [46].

High tertiles of vitamin E/total lipid ratio were not associated with decreased depressive symptoms in any age or sex group. Previous studies have suggested that vitamin E can lose its antioxidative properties and become pro-oxidative at high levels; high-dose vitamin E supplementation was found to cause increased oxidative stress [81,82]. As the depressive symptom levels of the high tertiles were neither decreased nor increased, their oxidative stress levels may have been similar to the reference group. We suggest that the vitamin E status of the high tertiles in the present study might have fallen around the threshold beyond which vitamin E becomes pro-oxidative.

In contrast to the findings of the main analysis, the exploratory analysis calculating odds ratios failed to reveal any significant association between vitamin E status and depressive symptoms. In this analysis, serum vitamin E levels were not adjusted for serum lipid levels, which could have led to error because a significant proportion of the participants had high lipid levels. Among all the participants, 39.8% had high total cholesterol levels (≥200 mg/dL) and 29.2% had high triglyceride levels (≥150 mg/dL). Because abnormal lipid levels can affect serum vitamin E levels, adjustment for total lipid levels is required in adults with hyperlipidemia [51]. In addition, 2 × 2 table analyses may not be sensitive enough to detect the subtle associations found in the main analysis, as the PHQ-9 score differences between vitamin E tertiles were relatively small. A recent study revealed a similar association between vitamin E and depression with the relationship discovered in the main analysis, providing support to the findings of the main analysis [83]. The study showed that increased vitamin E intake was associated with decreased rates of depression up until 15 mg/day, but additional intake above 15 mg/day did not change the odds of depression [83].

The present study discovered a non-linear relationship between vitamin E status and depressive symptoms. This could provide an explanation for the somewhat confusing results of several previous studies. Two prior studies reported decreased serum vitamin E levels in MDD patients compared to healthy individuals, but no significant correlation was found between serum vitamin E levels and depressive symptom severity among MDD patients [41,42]. Since correlation analyses assume linear relationships, it is possible that significant relationships might have been discovered if a statistical method suitable for detecting non-linear relationships had been used.

In the present study, educational attainment, equalized household income, residential area, living situation, smoking status, chronic medical disease, and physical activity showed significant associations with depressive symptoms. Previous studies have identified low educational attainment levels and income [54], living in a rural area [55], living alone [56], smoking [58], chronic disease [59], and inadequate physical activity [60] as risk factors for depression. The trends found in the present study were largely consistent with these findings.

Dietary supplement use was found to be associated with greater levels of vitamin E/total lipid ratio, suggesting that dietary measures may be helpful in correcting low vitamin E status. Therefore, dietary interventions in younger females and older males with a low vitamin E status could have a protective effect against depressive symptoms. This could potentially benefit many Korean adults, regarding their high rate of vitamin E deficiency [15]. Notably, studies with experimental designs have shown that vitamin E supplementation could significantly improve depressive symptoms [84], and a meta-analysis showed that dietary vitamin E intake is inversely associated with depression [33].

More than half of the present study’s sample used dietary supplements, which is consistent with a previous report in Korea [8]. This suggests that dietary supplements could be used as an effective tool for healthcare among Koreans. Based on the present study’s findings, Korean healthcare providers may screen depressed young females and older males for vitamin E deficiency, or screen vitamin-E-deficient young females and older males for depressive symptoms. In either way, confirmed vitamin E deficiency could be corrected by prescribing dietary supplements.

The strength of the present study is that it used a large and homogeneous nationwide dataset. In addition, the age- and sex-stratified approach enabled us to discover the different associations in each age and sex group. The four age and sex groups showed different baseline characteristics for every variable investigated, further supporting this stratification method. Moreover, the proper correction of serum vitamin E levels by serum lipid levels increased this study’s reliability, and the comparison between vitamin E/total lipid ratio tertiles enabled us to find a non-linear relationship. Nonetheless, the present study also has several limitations. First, causal relationships cannot be inferred from this study due to its cross-sectional design. In addition, it can be difficult to generalize the results of this study to clinically depressed individuals since hospitalized patients with severe depression were not included. Lastly, the discovered differences in depressive symptom severity depending on vitamin E status were relatively small. Greater effect sizes might have been detected if the antioxidant status derived from multiple types of antioxidants were analyzed, but we chose to focus on vitamin E due to the lack of data on other antioxidants.

## 5. Conclusions

In conclusion, the current study demonstrated that a low vitamin E status is associated with worse depressive symptoms in younger females and older males, independently of sociodemographic and clinical factors. In contrast, no such association was found in younger males or older females. A high vitamin E status was not associated with depressive symptoms regardless of age or sex. As dietary supplement use was associated with increased vitamin E status, younger females and older males with a low vitamin E status may benefit from dietary interventions to prevent depressive symptoms. Further longitudinal research should be conducted to determine the causality of the associations and the efficacy of dietary interventions. In addition, analyses using urinary metabolites of vitamin E and an integrated analysis of multiple antioxidants could be of interest for future studies.

## Figures and Tables

**Table 1:** Baseline characteristics of the study participants.

	Younger Participants (Age < 65)		Older Participants (Age ≥ 65)		
	Younger Males (*n* = 1551)	Younger Females (*n* = 1986)	Older Males (*n* = 432)	Older Females (*n* = 479)	*p*-Value
**Vitamin E, lipids, and PHQ-9 score**					
** Vitamin E (mg/L)**	13.16 ^a^ (4.71)	13.21 ^a^ (4.25)	13.10 ^a^ (4.98)	15.04 ^b^ (6.94)	**<0.001**
** Total cholesterol (mg/dL)**	192.80 ^ac^ (36.28)	194.61 ^a^ (36.36)	178.95 ^b^ (38.61)	189.35 ^c^ (39.31)	**<0.001**
** Triglyceride (mg/dL)**	169.94 ^a^ (157.67)	108.87 ^b^ (74.42)	139.25 ^c^ (80.32)	139.34 ^c^ (83.84)	**<0.001**
** Total lipid (mg/dL)**	669.91 ^a^ (201.51)	612.93 ^b^ (124.54)	607.76 ^b^ (134.74)	631.48 ^c^ (132.89)	**<0.001**
** Vitamin E/total lipid ratio (mg/g)**	1.99 ^a^ (0.54)	2.16 ^b^ (0.56)	2.16 ^b^ (0.66)	2.38 ^c^ (0.85)	**<0.001**
** PHQ-9 score**	1.99 ^a^ (3.12)	2.87 ^b^ (3.74)	2.11 ^a^ (3.63)	3.43 ^b^ (4.68)	**<0.001**
**Sociodemographic variables**					
** Educational attainment**					**<0.001**
Elementary school or below	86 (5.5)	170 (8.6)	175 (40.5)	334 (69.7)	
Middle school	106 (6.8)	188 (9.5)	72 (16.7)	75 (15.7)	
High school	580 (37.4)	741 (37.3)	125 (28.9)	48 (10.0)	
University or above	779 (50.2)	887 (44.7)	60 (13.9)	22 (4.6)	
**Equalized household income**					**<0.001**
Quartile 1 (low)	130 (8.4)	183 (9.2)	181 (41.9)	244 (50.9)	
Quartile 2	368 (23.7)	477 (24.0)	119 (27.6)	130 (27.1)	
Quartile 3	498 (32.1)	647 (32.6)	75 (17.4)	67 (14.0)	
Quartile 4 (high)	555 (35.8)	679 (34.2)	57 (13.2)	38 (7.9)	
**Residential area**					**<0.001**
Urban	1303 (84.0)	1688 (85.0)	326 (75.5)	357 (74.5)	
Rural	248 (16.0)	298 (15.0)	106 (24.5)	122 (25.5)	
**Living situation**					**<0.001**
Lives alone	150 (9.7)	146 (7.4)	63 (14.6)	144 (30.1)	
Lives with other household member(s)	1401 (90.3)	1840 (92.7)	369 (85.4)	335 (69.9)	
**Clinical variables**					
** Alcohol use status**					**<0.001**
No	392 (25.3)	1013 (51.0)	185 (42.8)	399 (83.3)	
Yes	1159 (74.7)	973 (49.0)	247 (57.2)	80 (16.7)	
** Smoking status**					**<0.001**
Non-smoker	982 (63.3)	1886 (95.0)	361 (83.6)	470 (98.1)	
Smoker	569 (36.7)	100 (5.0)	71 (16.4)	9 (1.9)	
** Chronic medical disease**					**<0.001**
None	1197 (77.2)	1557 (78.4)	134 (31.0)	90 (18.8)	
One	258 (16.6)	326 (16.4)	173 (40.1)	171 (35.7)	
Two or more	96 (6.2)	103 (5.2)	125 (28.9)	218 (45.5)	
** Physical activity**					**<0.001**
Inadequate	770 (49.7)	1076 (54.2)	248 (57.4)	346 (72.2)	
Adequate	781 (50.4)	910 (45.8)	184 (42.6)	133 (27.8)	
** BMI**					**<0.001**
Underweight	36 (2.3)	109 (5.5)	16 (3.7)	7 (1.5)	
Normal weight	466 (30.1)	991 (49.9)	154 (35.7)	144 (30.1)	
Overweight	393 (25.3)	383 (19.3)	112 (25.9)	132 (27.6)	
Obesity	656 (42.3)	503 (25.3)	150 (34.7)	196 (40.9)	

Continuous variables are presented as means and standard deviations; categorical variables are presented as numbers and percentages. Different superscripts signify significant mean differences. Statistically significant (*p* < 0.05) results are highlighted in bold. PHQ-9, Patient Health Questionnaire-9; BMI, body mass index.

**Table 2:** Linear regression analysis: association between vitamin E/total lipid ratio and depressive symptoms.

	PHQ-9 Score			
Vitamin E/Total Lipid Ratio	Model 1		Model 2	
	β	*p*-Value	β	*p*-Value
**Younger participants (Age < 65)**				
** Younger males**				
Tertile 1 (low)	0.01	0.834	−0.02	0.522
Tertile 2 (middle)	Ref		Ref	
Tertile 3 (high)	−0.01	0.668	0.00	0.911
** Younger females**				
Tertile 1 (low)	**0.08**	**0.004**	**0.07**	**0.008**
Tertile 2 (middle)	Ref		Ref	
Tertile 3 (high)	0.05	0.056	0.04	0.086
**Older participants (Age ≥ 65)**				
** Older males**				
Tertile 1 (low)	**0.16**	**0.004**	**0.13**	**0.018**
Tertile 2 (middle)	Ref		Ref	
Tertile 3 (high)	0.06	0.281	0.04	0.416
** Older females**				
Tertile 1 (low)	0.05	0.349	0.02	0.667
Tertile 2 (middle)	Ref		Ref	
Tertile 3 (high)	0.09	0.102	0.07	0.202

Model 1: Unadjusted; Model 2: Adjusted for educational attainment level, equalized household income, residential area, living situation, alcohol use status, smoking status, number of chronic medical diseases, adequacy of physical activity, and body mass index category. Statistically significant (*p* <0.05) results are highlighted in bold. PHQ-9, Patient Health Questionnaire-9.

## Data Availability

The data presented in this study are openly available on the KNHANES website http://knhanes.kdca.go.kr (accessed on 18 February 2023).

## Supplementary Materials

The following supporting information can be downloaded at: https://www.mdpi.com/article/10.3390/nu15081915/s1. Supplementary Table S1: The association between covariates and depressive symptoms in multivariable linear regression; Supplementary Figure S1: The proportions of vitamin E/total lipid ratio tertiles according to dietary supplement use status in (a) younger males, (b) younger females, (c) older males, and (d) older females.
