# Supplementary material for: Age and Sex Differences in the Association between Serum Vitamin E Levels and Depressive Symptoms: Korea National Health and Nutrition Examination Survey
Source: Nutrients. 2023 Apr 15;15(8):1915. doi: 10.3390/nu15081915 (PMC10144124; doi:10.3390/nu15081915)
Supplement: Supplementary file 1 [file nutrients-15-01915-s001.zip › nutrients-2326050-supplementary.pdf]

Supplementary material

Age and sex differences in the association between serum vitamin E levels and depressive symptoms

Hyunwoo Jeong [1], Jae Won Oh [2], Nak-Hoon Son [3], San Lee [2,4,*]

[1] Geumsan-gun Public Health Center, Geumsan 32726, Republic of Korea

[2] Department of Psychiatry, Yongin Severance Hospital, Yonsei University College of Medicine, Yongin 16995, Republic of Korea

[3] Department of Statistics, Keimyung University, Daegu 42601, Republic of Korea

[4] Department of Psychiatry and the Institute of Behavioral Science in Medicine, Yonsei University College of Medicine, Seoul 03722, Republic of Korea

* Correspondence: sanlee@yonsei.ac.kr

Supplemental Table S1. The association between covariates and depressive symptoms in multivariable linear regression

Covariates	Dependent variable: PHQ-9 score							
	Younger participants (Age <65)				Older participants (Age ≥65)			
	Younger male		Younger female		Older male		Older female	
	β	p-value	β	p-value	β	p-value	β	p-value
Sociodemographic variables								
**Educational attainment**								
Elementary school and below	Ref		Ref		Ref		Ref	
Middle school	**-0.09**	**0.009**	-0.02	0.584	-0.04	0.430	-0.04	0.466
High school	-0.09	0.118	-0.01	0.852	-0.06	0.277	-0.73	0.327
University or above	-0.09	0.123	0.00	0.993	0.04	0.523	-0.01	0.773
**Equalized household income**								
Quartile 1 (low)	Ref		Ref		Ref		Ref	
Quartile 2	**-0.18**	**<0.001**	**-0.15**	**<0.001**	-0.03	0.543	-0.01	0.859
Quartile 3	**-0.24**	**<0.001**	**-0.16**	**<0.001**	-0.11	0.051	**-0.14**	**0.008**
Quartile 4 (high)	**-0.27**	**<0.001**	**-0.22**	**<0.001**	**-0.13**	**0.021**	-0.06	0.204
**Residential area**								
Urban	Ref		Ref		Ref		Ref	
Rural	-0.03	0.214	**0.06**	**0.014**	0.03	0.571	-0.02	0.673
**Living situation**								
Lives alone	Ref		Ref		Ref		Ref	
Lives with other household members	**-0.09**	**<0.001**	-0.01	0.639	-0.08	0.096	-0.04	0.471
Clinical variables								
**Alcohol use status**								
No	Ref		Ref		Ref		Ref	
Yes	0.00	0.908	0.04	0.074	-0.03	0.353	0.00	0.998
**Smoking status**								
Non-smoker	Ref		Ref		Ref		Ref	
Smoker	**0.11**	**<0.001**	**0.15**	**<0.001**	0.01	0.860	0.05	0.265
**Chronic medical disease**								
None	Ref		Ref		Ref		Ref	
One	**-0.08**	**0.001**	0.04	0.068	-0.05	0.344	0.11	0.081
Two or more	**0.10**	**<0.001**	**0.10**	**<0.001**	**0.13**	**0.027**	**0.13**	**0.040**
**Physical activity**								
Inadequate	Ref		Ref		Ref		Ref	
Adequate	-0.01	0.729	0.03	0.176	**0.12**	**0.020**	-0.07	0.133
**BMI**								
Underweight	0.03	0.225	0.04	0.118	0.05	0.280	0.08	0.074
Normal weight	Ref		Ref		Ref		Ref	
Overweight	0.04	0.154	0.02	0.489	0.03	0.503	0.02	0.694
Obesity	0.01	0.711	-0.02	0.493	-0.05	0.315	-0.03	0.477

Statistically significant ($p < 0.05$) results are highlighted in bold.

PHQ-9, Patient Health Questionnaire-9; BMI, body mass index.

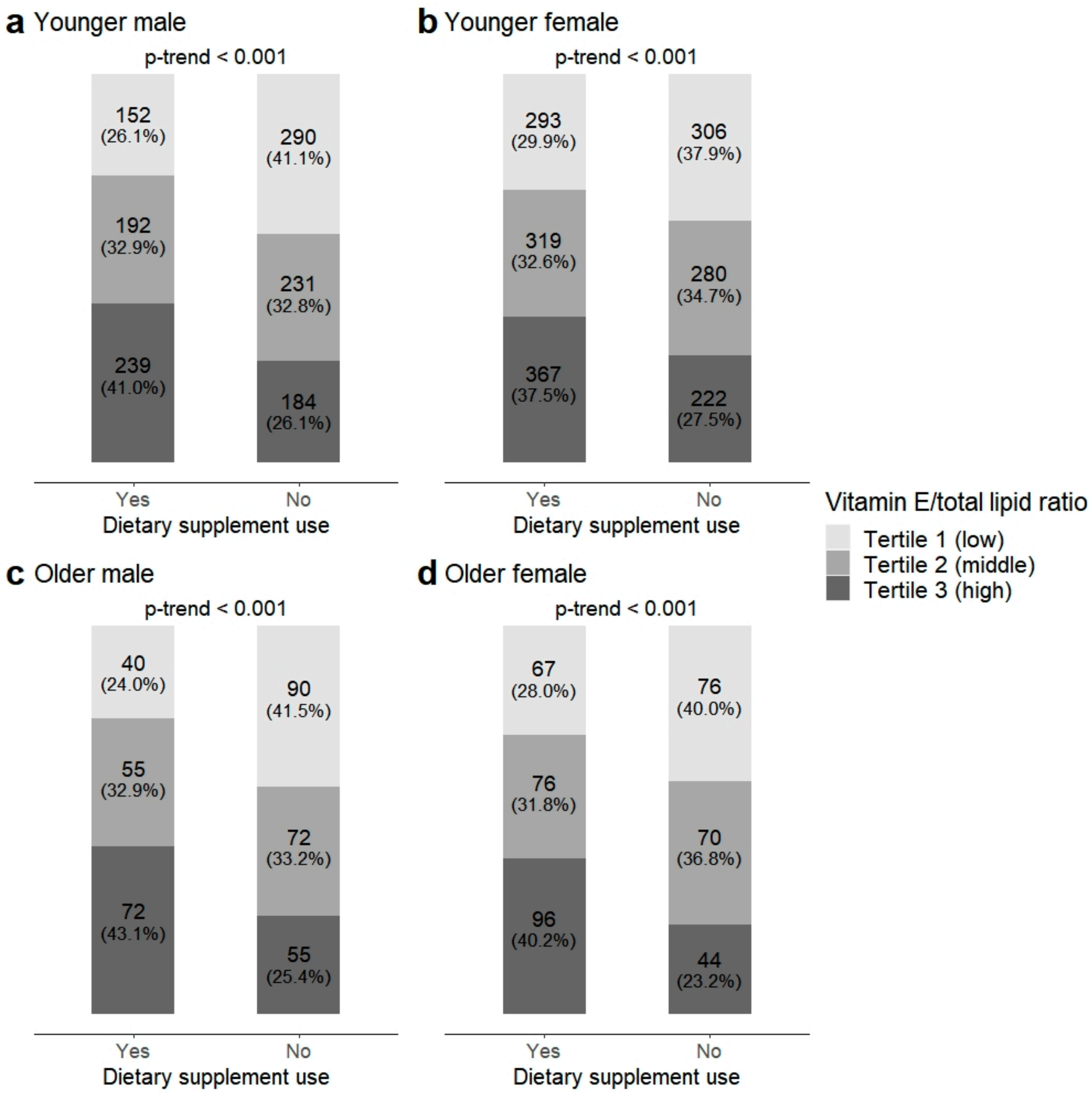

Supplemental Figure S1. The proportions of vitamin E/total lipid ratio tertiles according to dietary supplement use status in (a) younger males, (b) younger females, (c) older males, and (d) older females. Data are presented as numbers and percentages.
